# High levels of contamination and antimicrobial-resistant non-typhoidal *Salmonella* serovars on pig and poultry farms in the Mekong Delta of Vietnam

**DOI:** 10.1017/S0950268815000102

**Published:** 2015-03-17

**Authors:** L. T. P. TU, N. V. M. HOANG, N. V. CUONG, J. CAMPBELL, J. E. BRYANT, N. T. HOA, B. T. KIET, C. THOMPSON, D. T. DUY, V. V. PHAT, V. B. HIEN, G. THWAITES, S. BAKER, J. J. CARRIQUE-MAS

**Affiliations:** 1 Hospital for Tropical Diseases, Wellcome Trust Major Overseas Programme, Oxford University Clinical Research Unit, Ho Chi Minh City, Vietnam; 2 Centre for Tropical Medicine, Nuffield Department of Clinical Medicine, Oxford University, Oxford, UK; 3 Sub-Department of Animal Health Dong Thap, Cao Lanh, Vietnam; 4 London School of Hygiene and Tropical Medicine, London, UK

**Keywords:** Epidemiology, *Salmonella*, zoonoses

## Abstract

We investigated the prevalence, diversity, and antimicrobial resistance (AMR) profiles of non-typhoidal *Salmonella* (NTS) and associated risk factors on 341 pig, chicken, and duck farms in Dong Thap province (Mekong Delta, Vietnam). Sampling was stratified by species, district (four categories), and farm size (three categories). Pooled faeces, collected using boot swabs, were tested using ISO 6575: 2002 (Annex D). Isolates were serogrouped; group B isolates were tested by polymerase chain reaction to detect *S.* Typhimurium and (monophasic) serovar 4,[5],12:i:- variants. The farm-level adjusted NTS prevalence was 64·7%, 94·3% and 91·3% for chicken, duck and pig farms, respectively. Factors independently associated with NTS were duck farms [odds ratio (OR) 21·2], farm with >50 pigs (OR 11·9), pig farm with 5–50 pigs (OR 4·88) (*vs*. chickens), and frequent rodent sightings (OR 2·3). Both *S*. Typhimurium and monophasic *S.* Typhimurium were more common in duck farms. Isolates had a high prevalence of resistance (77·6%) against tetracycline, moderate resistance (20–30%) against chloramphenicol, sulfamethoxazole-trimethoprim, ampicillin and nalidixic acid, and low resistance (<5%) against ciprofloxacin and third-generation cephalosporins. Multidrug resistance (resistance against ⩾3 classes of antimicrobial) was independently associated with monophasic *S.* Typhimurium and other group B isolates (excluding *S*. Typhimurium) and pig farms. The unusually high prevalence of NTS on Mekong Delta farms poses formidable challenges for control.

## INTRODUCTION

Non-typhoidal *Salmonella* (NTS) is the generic name given to serovars belonging to *Salmonella enterica* subspecies I other than those that typically cause invasive disease in non-immunocompromised humans (i.e. *S*. Typhi and *S*. Paratyphi A). The majority of NTS infections do not result in clinical disease in animals. However, they are considered to be zoonotic and to have pathogenic potential in humans, typically causing self-limiting gastroenteritis [1]. In Vietnam NTS are responsible for up to 7% of cases of diarrhoea in children [2]. In addition, invasive infections due to NTS have become increasingly reported in Vietnam while infections due to *S.* Typhi are declining [3].

Due to economic and diagnostic limitations, full serotyping of NTS involved in disease in humans is not routinely performed in Vietnam, with most data on NTS serovar distribution originating from *ad hoc* research studies. For example, a study of 1419 paediatric cases of diarrhoea in 2009–2010 identified group B NTS in 58% of 77 identified NTS cases [4]. A previous study of 54 cases from five provincial hospitals in 2004 identified *S*. Typhimurium [phage type (PT) 90] as the most common serovar. The same study also identified *S*. Typhimurium PT90 as the second most common serovar in pigs after *S*. Anatum [5]. Monophasic *S*. Typhimurium (mST) variants (i.e. serovar 4,[5],12:i:-) lacking the first or the second phase H antigen due to a genetic deletion have been emerging since the 1990s worldwide, and are often associated with an increase in multidrug resistance (MDR) [6] but have not yet been reported in Vietnam.

A number of studies have investigated the presence of NTS in meat/carcasses in Vietnam [7–12]. However, to our knowledge, the farm-level prevalence of NTS and associated risk factors have not yet been systematically investigated in Vietnam; both of which are prerequisites for the development of control measures on farms.

We performed a survey on NTS in 341 pig and poultry farms in the Mekong Delta region of southern Vietnam using environmental faecal sampling methods. The aims of the study were: (1) to determine the prevalence and main serogroups of *Salmonella, S*. Typhimurium and mST in the main farmed species; (2) to describe antimicrobial resistance (AMR) patterns in NTS isolates; and (3) to investigate farm-level risk factors for NTS, *S*. Typhimurium, mST and MDR NTS.

## MATERIALS AND METHODS

### Survey design and data collection

The survey was conducted between February and May 2012 in Dong Thap (Vietnam), a Mekong Delta province characterized by high density of small-scale pig and chicken farms, as well as large numbers of duck flocks typically reared in synchronicity with the rice production cycle. The study covered 4/12 districts, three species (chicken, ducks, pigs) and three farm size categories. Farms were randomly selected from the district farm population census as previously described [13]. When a farmer refused participation, his farm was replaced by the next available one. Questionnaires (available upon request) were used to obtain basic farm demographics as well as other parameters related to the farmer and the farm management. Demographic statistical data pertaining to the commune (density of humans, pigs and poultry) were obtained from the Vietnamese Government Statistical Office (GSO).

### Sampling methods

We performed environmental sampling using swabs to collect naturally pooled faeces [14–16]. A fixed number of boot swabs were collected from small (*n* = 3), medium (*n* = 4) and large (*n* = 5) farms. This sampling scheme was formulated as a compromise between sensitivity, simplicity and the requirement for standardization. Since one pair of boot swabs is roughly equivalent to sampling 60 individual faecal droppings [17], we assumed that our sampling method was equivalent to sampling a constant proportion of animals from each farm. Briefly, areas dedicated to housing the farms' target species were divided into equally sized sections. From each section a pair of boot swabs was taken by walking over 50 steps with a shuffling motion focused on areas where fresh droppings were visible. Where walking on housing areas was not possible (i.e. sows in crates/farrowing pens, chickens in cages or stilt houses), hand-held gauze swabs were used to sample faeces from 10 sampling points (~25 g per swab). Recent studies have shown that three pairs of boot swabs used per flock are equivalent to sampling methods used in the European Union NTS surveys [18].

### Laboratory methods

Samples were immediately sent to the laboratory under refrigeration (4–8 °C). Cultures were initiated within 12 h of collection. The testing method was a modified version of ISO 6579:2002 (Annex D), involving: (*a*) pre-enrichment in buffered peptone water (BPW) (37 °C, 18 h); (*b*) plating on modified semi-solid Rappaport–Vassiliadis (MSRV) (41 °C, 24 h) and (*c*) plating onto Rambach agar (37 °C, 24 h) [19]. Suspect NTS colonies were confirmed by serogrouping based on the Kauffmann–White typing scheme using relevant poly O antiserum [20].

Only one colony was randomly selected from each swab sample. Isolates were classified as either group B, C, D, or ‘other’ NTS. Confirmed NTS group B isolates were further tested by reverse transcriptase–polymerase chain reaction (RT–PCR) for *S. Typhimurium* and mST (serovar 4,[5],12:i:-) by conventional PCR [21]. All isolates were tested for antimicrobial susceptibility against 10 antimicrobials belonging to seven classes using the Kirby–Bauer (disk diffusion) method on Muller–Hinton Agar (Oxoid, UK). The antimicrobials and concentrations were as follows: (1) penicillins (with and without clavulanic acid): ampicillin (AMP, 10 *μ*g) and amoxicillin plus clavulanic acid (AUG, 30 *μ*g); (2) third-generation cephalosporins: ceftriaxone (CRO, 30 *μ*g), and ceftazidime (CAZ, 30 *μ*g); (3) phenicols: chloramphenicol (30 *μ*g); (4) quinolones: nalidixic acid (NA, 30 *μ*g) and ciprofloxacin (CIP, 5 *μ*g); (5) aminoglycosides: gentamicin (CN, 10 *μ*g); (6) potentiated sulphonamides: sulfamethoxazole-trimethoprim (SXT); (7) tetracyclines: tetracycline (TE, 30 *μ*g). Results were interpreted according to breakpoints as defined by the Clinical and Laboratory Standards Institute (CLSI) guidelines for Enterobacteriaceae [22].

Isolates were assigned to a serovar by multilocus sequence typing (MLST) on DNA extracted using the Wizard Genomic DNA extraction kit (Promega, USA) from one full loop of pure NTS colonies of overnight culture in nutrient agar media. PCR amplification was performed on the seven MLST loci *aro*C, *dna*N, *hem*D, *his*D, *pur*E, *suc*A and *thr*A, as described previously [22]. PCR products were purified using QIAquick column-based PCR purification system (Qiagen, USA) and sequenced in forward and reverse directions using the Big Dye Cycle Sequencing kit (Applied Biosystems, USA) on an ABI 3770 automatic sequencer according to the manufacturer's instructions. After generating a sequence type, strains were inferred to serotypes according to the data available on the MLST database (http://mlst.warwick.ac.uk/mlst/dbs/Senterica/).

Due to economic limitations, we performed selective MLST-based serovar identification on one isolate representing each phenotypic pattern, defined by the combination of (*a*) serovar/serogroup (*S*. Typhimurium, mST, other group B, group C, group D, and ‘other group’); and (*b*) sensitivity results for 10 antimicrobials. The tested isolate was chosen at random.

### Statistical analyses

Data from all farms were combined into a unique dataset aiming to model the overall probability of a swab testing positive for NTS. Since the number of farms in each stratum (*i* = 1, …, 36) was known from census data, prevalence estimates and odds ratio (OR) estimates, regression models were adjusted by assigning a stratum-specific sampling weight (*W*_*i*_) to each observation unit (swab):

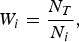

where *N*_T_ is the total number of farms ( 35 248) and *N*_*i*_ is the number of farms sampled in each stratum. Standard errors were corrected to take into account potential similarities between farms in each stratum [23]. The prevalence of a positive boot swab per farm was compared for each species. Risk factor analyses at swab level were performed using hierarchical logistic regression with ‘farm’ as the clustering variable for the following outcomes: (1) prevalence of NTS; (2) prevalence of *S*. Typhimurium-positive results (*vs*. any other result, including negative); and (3) prevalence of mST result (*vs*. any other result, including negative).

Each NTS isolate was characterized by a profile defined by its unique combination of type (*S*. Typhimurium, mST, other group B, group C, group D, other group) and its susceptibility/resistance against the 10 antimicrobials tested. Shannon–Weaver entropy (*H*) was used to estimate the farm-level diversity of isolates from the same farm. *H* was defined as:

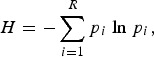

where *p*_*i*_ is the proportion of isolates with the *i*th resistance profile, and *R* is the total number of profiles. Higher values of *H* correspond to greater diversity in AMR profiles.

The prevalence of AMR against all antimicrobials tested (and their corresponding 95% confidence intervals; CI) was calculated for *S*. Typhimurium, mST and other group B serovars, groups C, and D, and other group serovars separately. Hierarchical logistic regression models with the variable ‘farm’ as a random effect were built using data on all NTS isolates to investigate the outcome of ‘probability of a multidrug resistant (MDR) strain’, defined as resistance against ⩾3 classes of antimicrobials. Variables investigated in all models were: (1) type of farm, defined by the target species and size (nine categories); (2) age, gender and years of experience of the farmer; (3) use of antimicrobials on the farm over the last 3 months; (4) use of any *Salmonella* vaccines on target species on farm; (5) history of disease on farms (enteric, respiratory or other); (6) presence of chickens/ducks/pigs as ‘non-target species’; (7) presence of cattle/buffalo/dog/cat; (8) type of water (well, municipal); and (9) frequency of rodent and wild bird sightings. In all models candidate variables were those significant (<0.05) in the univariable models for any of the three outcomes. Variables were ranked by their degree of significance and were included in the model starting with the most significant ones using a step-wise forward approach [24]. In each of the final multivariable models, variables were retained if their *P* value was <0·05 for any of the outcome variables. All interactions between significant variables in the model were assessed. The suitability of each new variable included in the model was assessed using the Wald test [23]. All interactions between final significant variables were tested. All statistical analyses were performed using R packages ‘survey’ and ‘epicalc’ (http://www.r-project.org).

## RESULTS

The total number of farms in the sampling frame as well as the actual number of farms sampled (sampling units) and their associated sampling weights are shown in Supplementary Table S1. A total of 341 farms were sampled (117 chicken, 118 duck, 104 pig farms) using a total of 1349 swabs, and 739 NTS isolates were recovered. Since small chicken farms were by far the predominant farm category (66·2% of all farms), swabs from these farms were assigned the largest sampling weight.

### Farm-level prevalence

**Fig. 1.**
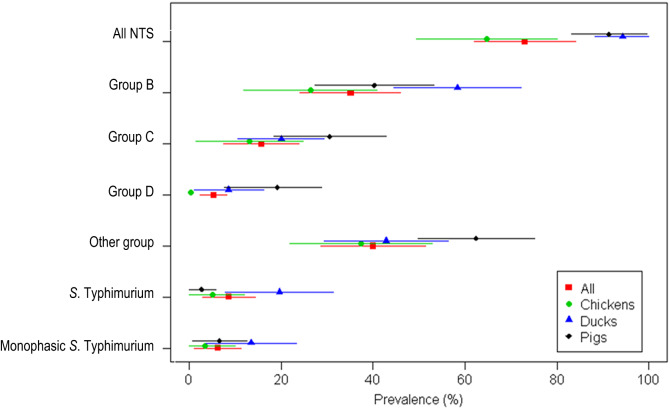
Farm-level prevalence of non-typhoidal *Salmonella* (NTS) adjusted for the sampling frame, by host species (chickens, ducks, pigs) (lines indicate 95% confidence intervals) (Dong Thap, Mekong Delta, 2012).

The crude farm-level prevalence by species is shown in Table 1. The overall survey adjusted farm-level prevalence of NTS was 73·0% (95% CI 62·0–84·0) (Fig. 1). The highest survey-adjusted NTS prevalence corresponded to duck farms (94·3%, 95% CI 88·3–100·0), followed by pig farms (91·3%, 95% CI 83·1–99·5) and chicken farms (64·7%, 95% CI 49·4–80·0). Chicken farms had a significantly lower NTS prevalence than both duck and pig farms (*P* < 0·05). There were no statistically significant differences in prevalence by district for any of the three species (data not shown).

**Table 1.** Crude farm-level prevalence by farm category (Dong Thap, Mekong Delta, 2012)

Target species	No. farms	Small		Medium		Large	
		No. positive/ total	Prevalence	No. positive/ total	Prevalence	No. positive/ total	Prevalence
Chicken	117	22/36	61·1%	24/42	57·1%	28/39	71·8%
Duck	118	32/35	91·4%	45/50	90·0%	33/35	94·3%
Pig	104	34/37	91·9%	32/38	84·2%	25/29	86·2%
	341	88/108	81·5%	101/130	79/6%	86/103	84·2%

*S*. Typhimurium was most commonly isolated from duck farms (19·6%) and least from pig farms (2·7%). mST was most commonly isolated from duck farms (13·5%). The farm-level prevalence of NTS belonging to groups other than B, C, or D was 62·4% (pig farms), 42·8% (duck farms), and 37·3% (chickens farms).

### Sample-level prevalence

**Fig. 2.**
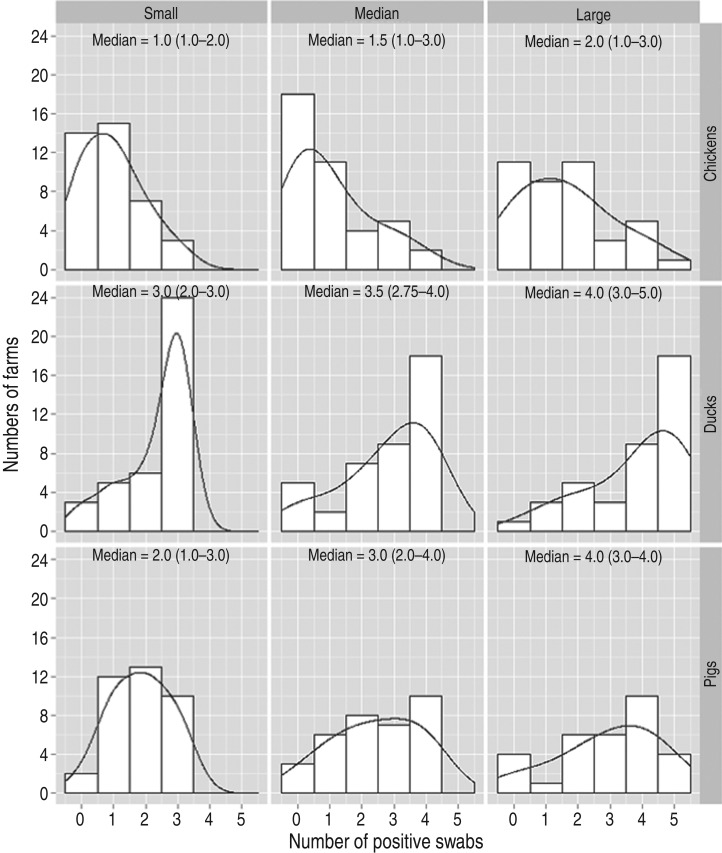
Distribution of farms by number of boot swabs positive for non-typhoidal *Salmonella*, presented separately by host species and size (Dong Thap, Mekong Delta, 2012).

At the sample (swab) level, the highest prevalence of NTS corresponded to samples from duck farms (69·8%, 95% CI 61·2–76·3), followed by pig (65·4%, 95% CI 59·0–71·9) and chicken (31·0%, 95% CI 22·3–39·3) farms (Fig. 2).

### Diversity of NTS profiles by farm type

Of farms with at least two NTS isolates, the overall *H* was greatest for pig farms [median 0·69, interquartile range (IQR) 0·56–1·1], followed by duck (median 0·69, IQR 0·56–1·0) and chicken (median 0·61, IQR 0–0·69) farms, although the difference between different species was not statistically significant (Kruskal–Wallis *χ*^2^ = 3·44, *P* = 0·178). Of all farm types, large duck farms had the greatest diversity of profiles (*H* = 0·74, IQR 0·69–1·1).

### Risk factors for NTS, S. Typhimurium and mST

The nine farm-type categories were combined into four groups, (*a*) chicken farms (baseline); (*b*) duck farms; (*c*) small pig farms; and (*d*) medium and large pig farms, given the observed statistically significant differences in risk between (*b*), (*c*), and (*d*) and baseline. Swabs from duck farms were associated with a higher prevalence of NTS compared to chicken swabs (OR 21·2). Compared to chicken farms, pig farms had an increased risk, although the magnitude of risk was higher for large farms (OR 11·9, compared to OR 4·9 for smaller pig farms). Other factors associated with NTS were ‘rodents seen at least weekly’ (OR 2·3), and male farmer (OR 2·5). The interaction between ‘high frequency of rodent sightings’ and farm type indicated an additional increase in risk for small pig farms, large pig farms and chicken farms with high frequency of rodent sightings (OR 8·8, 21·3, and 1·8, respectively) (compared to chicken farms with low frequency of rodent sightings). By contrast, duck farms with a high frequency of rodent sightings had no increased risk (*vs*. duck farms with low frequency of rodent sightings) (OR 0·79, *P* = 0·26).

Of the three species, ducks had the greatest prevalence of *S*. Typhimurium, although it was only significantly greater compared to pigs (but not chickens). The human density at commune level was a strong protective factor for *S*. Typhimurium (log number of humans/km^2^, OR 0·5).

Duck flocks had a greater probability of testing positive with mST compared to chickens or pigs (OR 6·8), as did farms located in areas of high density of pig farms (OR 1·6). By contrast, supplying animals with municipal water was a strong protective factor (OR 0·1) for all species (Table 2).

**Table 2.** Significant risk factors for NTS, *S.* Typhimurium and monophasic *S.* Typhimurium at sample level (Dong Thap, Mekong Delta, 2012)

	OR	95% CI	*P* value
**Model A: Outcome NTS (any)**			
Animal species (baseline = chicken farm)	1·0	—	—
Pig farm 5–50 pigs	4·88	2·83–8·43	<0·001
Pig farm >50 pigs	11·87	4·20–33·53	<0·001
Duck farm	21·17	8·39–53·46	<0·001
Rodents seen at least weekly	2·29	1·06–4·95	0·034
Male farmer	2·47	1·05–5·81	0·039
Interaction rats seen at least weekly × duck farm	0·26	0·09–0·76	0·015
**Model B: Outcome *S.* Typhimurium**			
Male farmer (baseline = female)	3·03	1·90–4·86	<0·001
Target species (baseline = pig farm)	1·0	—	
Chicken	2·50	0·38–15·9	
Duck	7·44	2·0–27·6	
log(human density*)	0·50	0·31–0·78	0·002
**Model C: Outcome monophasic *S*. Typhimurium**			
Duck target spp. (*vs*. chicken and pig)	6·76	1·07–42·5	0·042
Municipal water	0·07	0·01–0·38	0·002
log(density of pig farms†)	1·56	1·18–2·06	0·002

NTS, Non-typhoidal *Salmonella*; OR, odds ratio; CI, confidence interval.

Intercept model 1 (2·60, s.e. = 1·21); intercept model 2 (−4·87, s.e. = 1·62); intercept model 3 (−6·25, s.e. = 1·25);

* No. of humans/km^2^ in commune.

† No. of pig farms/km^2^ in commune.

A total of 97/103 pig farmers reported that they had vaccinated their herds against *Salmonella* over the previous year, using both inactivated and attenuated preparations containing *S*. Cholerasuis and/or *S*. Typhimurium strains. There was no difference in prevalence between vaccinated and unvaccinated herds (data not shown). None of the chicken and duck farms reported using *Salmonella* vaccines.

### Antimicrobial resistance

A total of 727/739 (98·4%) NTS isolates (50 *S*. Typhimurium, 45 mST, 168 other group B, 101 group C, 53 group D, 310 ‘other’ groups) were tested for AMR. The highest observed levels of resistance were against tetracyclines (77·6%) chloramphenicol (27·2%), sulfamethoxazole-trimethoprim (27·0%), ampicillin (24·2%), and nalidixic acid (22·8%). The proportion of strains resistant against ciprofloxacin was 1·3%. The proportion of MDR isolates (resistant to ⩾3 classes of antimicrobials) was 30·1%. The proportion of MDR was greatest for group B serovars other than *S*. Typhimurium and mST (42·8%), followed by mST (37·8%), and *S*. Typhimurium (22·0%). MDR was lowest for groups C and D NTS (11·9% and 9·4%, respectively) (Table 3). Compared to poultry NTS isolates, pig isolates had higher levels of AMR against tetracycline (83·3% *vs*. 68·1–77·6%), ampicillin (41·3% *vs*. 14·3–19·4%), chloramphenicol (41·3% *vs*. 20·1–20·4%), gentamicin (7·5% *vs*. 2·6–4·2%) and sulfamethoxazole-trimethoprim (36·7% *vs*. 14·6–25·4%). NTS isolates resistant to third-generation cephalosporins (ceftriaxone and/or ceftazidime) were identified in all three animal species, although at low numbers (<1%) (data not shown).

**Table 3.** Percentage of non-typhoidal *Salmonella* isolates (n = 727) resistant and multi-drug resistant (MDR) tested against a panel of 10 antimicrobials (Dong Thap, Mekong Delta, 2012)

	All serovars	*S*. Typhimurium	Monophasic *S*. Typhimurium	Other group B	Group C	Group D	Other groups
AMP	24·2 (21·1–27·3)	22·0 (10·5–33·5)	57·8 (43·3–72·2)	20·8 (14·7–27·0)	12·9 (6·3–19·4)	9·4 (1·6–17·3)	27·7 (22·8–32·7)
AUG	5·1 (3·5–6·7)	4·0 (0·0–9·4)	13·3 (3·4–23·3)	1·8 (0·0–3·8)	3·0 (0·0–6·3)	1·0 (0·0–3·7)	7·4 (4·5–10·3)
CRO	0·7 (0·0–1·3)	0·1 (0·0–1·0)	4·4 (0·0–10·5)	0·6 (0·0–1·8)	0·1 (0·0–0·7)	0·1 (0·0–1·0)	0·6 (0·0–1·5)
CAZ	0·6 (0·0–1·1)	0·1 (0·0–1·0)	4·4 (0·0–10·5)	0·1 (0·0–0·6)	0·1 (0·0–0·7)	0·1 (0·0–1·0)	0·6 (0·0–1·5)
TE	77·6 (74·5–80·6)	86·0 (76·4–95·6)	86·7 (76·7–96·6)	87·5 (82·5–92·5)	65·3 (56·1–74·6)	67·9 (55·4–80·5)	75·2 (70·4–80·0)
C	27·2 (24·0–30·5)	18·0 (7·4–28·6)	35·6 (21·6–49·5)	39·9 (32·5–47·3)	5·0 (0·7–9·2)	0·1 (0·0–1·0)	32·6 (27·4–37·8)
NA	22·8 (19·8–25·9)	12·0 (3·0–21·0)	26·7 (13·7–39·6)	37·5 (30·2–44·8)	8·9 (3·4–14·5)	9·4 (1·6–17·3)	22·9 (18·2–27·6)
CIP	2·8 (1·6–3·9)	0·1 (0·0–1·0)	8·9 (0·6–17·2)	6·0 (2·4–9·5)	0·1 (0·0–0·7)	0·1 (0·0–1·0)	1·9 (0·4–3·5)
CN	4·5 (3·0–6·1)	6·0 (0·0–12·6)	20·0 (8·3–31·7)	7·1 (3·2–11·0)	0·1 (0·0–0·7)	0·1 (0·0–1·0)	2·9 (1·0–4·8)
SXT	27·0 (23·7–30·2)	26·0 (13·8–38·2)	37·8 (23·6–51·9)	36·9 (29·6–44·2)	8·9 (3·4–14·5)	0·1 (0·0–1·0)	30·6 (25·5–35·8)
MDR	30·1 (28·4–31·8)	22·0 (10·5–33·5)	37·8 (23·6–51·9)	42·9 (35·4–50·3)	11·9 (5·6–18·2)	9·4 (1·5–17·3)	32·9 (27·7–38·1)

Values given are % (95% confidence interval).

AMP, ampicillin; AUG, amoxicillin/clavulanic acid; CRO, ceftriaxone; CAZ, ceftazidime; TE, tetracycline; C, chloramphenicol; NA, nalidixic acid; CIP, ciprofloxacin; CN, gentamicin, SXT, sulfamethoxazole-trimethoprim.

### Risk factors for MDR

MDR was associated with pig farms (*vs*. other species) (OR 4·5) and with mST (OR 18·5), other group B serovars (OR 13·6), groups other than B, C, or D (OR 6·3), and *S*. Typhimurium (OR 6·2) (*vs*. groups C and D). A high density of pig farms in the commune was also a risk. The calculated OR of 1·4 is equivalent to an OR of 6·8 for a farm located in a commune with a density of 86 pig farms/km^2^ (75% quantile) *vs*. farms in communes with density of 16 pig farms/km^2^ (25% quantile). The human density was not associated with increased resistance and was therefore excluded from the model (Table 4).

**Table 4.** Results of multivariable-level model investigating risk factors for MDR in 727 NTS isolates (Dong Thap, Mekong Delta, 2012)

Variable	Level	OR	95% CI	*P* value
Animal species	Baseline = chicken and duck farm	1·0	—	—
	Pig farm	4·53	2·17–9·46	<0·001
Type of NTS	Baseline = groups C and D	1·0	—	—
	Monophasic *S*. Typhimurium	18·53	4·89–70·24	<0·001
	*S*. Typhimurium	6·23	1·67–23·26	0·003
	Other group B	13·56	5·12–35·87	0·0 001
	Other groups*	6·35	2·62–15·37	0·001
log(density of pig farms†)		1·45	1·10–1·91	0·0 023

MDR, Multidrug resistance; NTS, non-typhoidal *Salmonella*; OR, odds ratio; CI, confidence interval.

Model intercept: −5·38 (s.e. = 0·76).

* Isolates belonging to groups other than B, C, or D.

† No. of pigs/km^2^ in the commune;

### Serovar allocation to representative AMR/serogroup patterns by MLST

Of isolates other than *S*. Typhimurium and mST (*n* = 632) there were 63 unique patterns reflecting the combinations of NTS group (groups B, C, D, and other) and AMR test result. A total of 14 different serovars were identified by testing one randomly selected isolate within each pattern. A total of 391/632 isolates (61·9%) corresponded to seven patterns of isolates either fully susceptible, or tetracycline-only resistant: group B fully susceptible (*n* = 18, assigned as Paratyphi B var. Java); group B tetracycline resistant (*n* = 71, *S*. Derby); group C fully susceptible (*n* = 31, *S*. Rissen), group C tetracycline-resistant (*n* = 48, *S*. Bareilly); group D fully susceptible (*n* = 17; *S*. Dublin); ‘other group’ fully susceptible (*n* = 67, *S*. London); ‘other group’ tetracycline-resistant (*n* = 108, *S*. Weltvreden).

The remaining 56 patterns (i.e. with resistance pattern other than tetracycline-only), representing 241 isolates, and their allocated serovar identity are presented in Table 5. The most commonly identified serovars were *S*. Derby (*n* = 9 patterns), *S*. London (*n* = 7), *S*. Anatum (*n* = 7), *S*. Indiana (*n* = 6), and *S*. Rissen (*n* = 5).

**Table 5.** Serovar identity assigned by MLST to individual serogroup resistance patterns (Dong Thap, Mekong Delta, 2012) (N = 56 patterns). Fully susceptible or tetracycline resistant-only patterns were excluded

MLST-assigned serovar	Pattern	No. of isolates	No. of farms where present		
			Chicken	Duck	Pig
Derby	Other group B: AMP-C-NA-SXT	1			1
	Other group B: AMP-TE-C-NA-CIP-SXT	1		1	
	Other group B: AMP-TE-C-NA-CN-SXT	3		1	2
	Other group B: AMP-TE-C-NA-SXT	9	1	3	
	Other group B: AMP-TE-C	8	1		4
	Other group B: TE-C	3			3
	Other group B: TE-NA	2		1	1
	Other group B: TE-C-NA	3	1		2
Indiana	Other group B: AMP-AUG-TE-C-NA-CIP-CN-SXT	2		1	
	Other group B: AMP-TE-C-NA-CIP-CN-SXT	7		2	4
	Other group B: AMP-AUG-TE-C-NA-SXT	1		1	
	Other group B: TE-C-NA-SXT	24		10	
	Other group B: TE-NA-SXT	9	1	3	
	Other group B: NA-SXT	1	1		
Paratyphi B var. Java monophasic	Other group B: CRO-C	1	1		
Bareilly	Group C: AMP-AUG-TE-NA	1			1
	Group C: TE-SXT	1			1
Hadar	Group C: AMP-AUG-TE	2		1	1
	Group C: TE-NA	2	1		1
	Group C: NA	3	1	1	1
Litchfield	Group C: AMP-TE-NA	1	1		
Newport	Group C: TE-C-SXT	2		2	
	Group C: AMP-TE-C-SXT	4			3
Rissen	Group C: AMP-TE-C-NA-SXT	2			2
	Group C: AMP-TE-SXT	4			4
	Group C: AMP-TE	2	2		
	Group C: TE-C	1			1
	Group C: AMP	1			1
Enteritidis	Group D: AMP-TE-NA	5	1	2	
Anatum	Other: AMP-TE-NA-SXT	1			1
	Other: AMP-TE-SXT	3			3
	Other: TE-C-SXT	10	1	2	5
	Other: TE-C-NA	4	2	1	1
	Other: TE-C	11	5		4
	Other: TE-C-NA-SXT	2		1	
	Other: TE-NA-SXT	2	1	1	
Give	Other: AMP-AUG-CRO-CAZ-TE-C-NA-SXT	1			1
	Other: AMP-TE-C-NA-CIP-CN-SXT	1			1
	Other: AMP-TE-C-NA-CIP-SXT	2	1		1
London	Other: AMP-AUG-TE-C-NA-SXT	15	2	7	2
	Other: AMP-AUG-TE-NA	1		1	0
	Other: AMP-TE-C-NA-SXT	23	5	3	10
	Other: AMP-TE-C-SXT	21		3	10
	Other: -CRO-TE-NA	1		1	
	Other: -TE–NA-CIP–SXT	1		1	
	Other: -TE-NA-SXT	2		1	
	Other: C-SXT	2	1		1
	Other: TE-NA	7		3	3
	Other: NA	5		4	1
	Other: AMP-C	1		1	
Senftenberg	Other: AMP-TE-C-CN-SXT	6	5	1	
Weltvreden	Other: AMP-AUG-TE-C-NA-CIP-CN-SXT	2			1
	Other: AMP-AUG-TE	4	2	1	1
	Other: AMP-TE	5		1	4
	Other: CAZ	1		1	
	Other: NA-SXT	1		1	

MLST, Multilocus sequence typing.

In chicken farms, patterns assigned to *S*. London and *S*. Anatum were predominant; in duck farms *S*. Indiana and *S*. London; and in pig farms *S*. Derby and *S*. London were predominant.

## DISCUSSION

Results from this study indicate an unusually high farm-level prevalence of NTS (73%) in the Mekong Delta of Vietnam, the highest (>90%) corresponding to pig and duck farms. Farms surveyed in the European Union (EU) using standardized methodologies (2005–2008) had a marked overall lower prevalence, although NTS was more common in pig farms (33·3%) compared to chicken broiler and layer farms (23·7 and 30·8%, respectively) [25–27].

In our survey duck farms had the highest farm-level NTS prevalence (94·3%). A possible explanation is the relatively less attention to biosecurity and the characteristic management system of duck flocks that involve grazing on rice fields [28]. A previous study also reported a higher animal-level prevalence in ducks (8·7%) compared to pigs and chickens (5·2–7·9%) in the Mekong Delta, although these differences were not significant [29]. Data from Denmark (2012), where duck flocks are routinely monitored, indicate a higher prevalence of NTS infection in duck flocks compared to chicken flocks (46·0% *vs*. 0·8%) [30]. Our data also indicated that, of the three species, ducks also had the highest *per sample* prevalence. This probably reflects higher levels of animal-level infection, since the sensitivity of pooled samples is a function of within bird-level prevalence [31].

A high frequency of rat sightings on farms was a significant risk factor. Studies have documented an association between rodents and increased risk of NTS on farms [32, 33]. Rats are extremely prevalent in the Mekong Delta. A study on 600 rats trapped in several Mekong Delta provinces indicated high NTS prevalence (19·3%) (40% for rats trapped in Dong Thap). A total of 94% rats were colonized by either NTS serovars London, Weltevreden or Derby, all identified in our study [34]. Most farm buildings in the Mekong Delta are not rat-proof, and pest control measures are normally insufficient. In these circumstances rodents can easily access feed storages where they often defecate. This may plausibly contribute to infection of farmed animals through consumption of contaminated feed. The significant (protective) interaction between ducks and rodents suggests that, unlike in the case of pigs and chickens, rats probably play a lesser role in transmission of NTS to ducks, and other sources linked to their grazing practices may be more relevant. However the low levels of biosecurity in Mekong Delta farms suggest the potential role of many potential vectors and reservoirs.

We report a higher risk in larger pig farms (*n* > 50), in agreement with previous studies [35, 36], reflecting the challenge in controlling NTS in pig production, probably due to the continuous introduction of new NTS strains via replacement of breeding stock, as well as the intrinsic difficulties of adequately disinfecting occupied buildings.

Although most NTS infections in animals are asymptomatic, some host species-adapted serovars such as *S*. Cholerasuis and *S.* Typhisuis (pigs) and *S*. Pullorum and *S*. Gallinarum (chickens) often cause fatal disease [37]. In pigs, septicaemic episodes of *S*. Typhimurium have been reported [38]. In Mekong Delta pig farmers there is a strong perception that NTS infections cause significant production losses, and this explains the high proportion of farm owners reporting routine vaccination (94%).

Of the three animal species investigated, serovars *S*. Typhimurium and mST were more common in ducks. This is in contrast with European countries where these serovars are predominantly isolated from pig farms and pork [25, 39, 40]; however, the size of the duck industry in the EU is very small and data on NTS prevalence in ducks is limited.

The negative association between mST and municipal water suggests that water sources other than municipal water (i.e. river, well or rain water) may represent potential risks, although no specific water source categories remained significant in the multivariable models.

With the exception of tetracyclines, against which most (77·8%) isolates were resistant, levels of resistance against ampicillin, chloramphenicol, and nalidixic acid were generally lower (22·8–27·0%) compared to NTS isolates from meat samples in northern Vietnam (24·5–39·8%) [12, 41]. We found a strong association between group B serovars and AMR, independent of the animal host species, with the highest prevalence of MDR corresponding to mST, followed by ‘other’ group B serovars.

Compared to chicken and duck isolates, a higher proportion of NTS isolates from pigs were antimicrobial resistant. In addition AMR was partly explained by the density of pig farms, suggesting that environmental contamination with antimicrobials or other AMR organisms from pig farms in the vicinity could have an impact beyond the pig farms themselves. Nevertheless, our results are in agreement with routine monitoring data collected in the EU where resistance levels in isolates from pigs against tetracyclines, ampicillin and sulphonamides were higher than in broiler isolates (55–59% *vs*. 20–27%). By contrast, EU chicken NTS isolates had moderate to high levels of resistance against nalidixic acid and ciprofloxacin (23–24%), whereas pig isolates had low levels of resistance (2–3%) [42]. However, surveillance data on AMR from animal NTS in the EU indicate resistance based on EUCAST epidemiological cut-off values, not clinical breakpoints. Because of this, the observed differences should be viewed with caution.

NTS isolates had low levels of AMR (<1%) against third-generation cephalosporins, in agreement with previous findings [9, 12, 43], and in contrast with data from Malaysia, Singapore and Thailand (>3%) [44].

The emergence of mST worldwide has been associated with clonal groups resistant to ampicillin, sulphonamides, streptomycin and tetracyclines [45, 46]. Although we did not test for streptomycin resistance, our results confirm a high proportion (31·1%) of mST co-resistant against ampicillin, sulphonamides and tetracyclines, *vs.* 16·9% in other serovars (data not shown).

We used MLST to test isolates representing unique serogroup/AMR combinations, excluding fully susceptible or tetracycline resistant-only isolates. Unfortunately, we could not perform MLST testing on all 739 isolates due to economic limitations. Clearly testing was insufficient for the (more common) fully susceptible or tetracycline-only susceptible serovars. It is expected that more economic alternatives to conventional serotyping will become available in developing countries to enable the characterization of NTS on farms and the investigation of human outbreaks [39].

In summary, we demonstrated an exceptionally high prevalence and high diversity in NTS serovars across farms in the Mekong Delta. Of the three species investigated, ducks had the highest NTS prevalence although pigs were associated with the highest levels of MDR. Levels of AMR were considerably high for most antimicrobials investigated, except for amoxicillin/clavulanic acid, ciprofloxacin and third-generation cephalosporins.

Our study confirmed the widespread presence of MDR mST in Mekong Delta farms, as reported in other countries. Farming practices in the Mekong Delta are typical of many developing countries in the region. There is a concern that fast increases in animal production are not matched by increases in biosecurity and testing programmes for foodborne pathogens. In Vietnam, virtually no NTS serotyping is conducted in human or animal diagnostic laboratories. However, given the country's rapid economic development and the increasing potential for export markets this may soon change. The unusually high prevalence, the predominance of mixed-species farming without adequate biosecurity, and the abundance of vectors (rats) suggest that control of NTS on farms in the Mekong Delta of Vietnam will be particularly challenging.

## Supplementary material

For supplementary material accompanying this paper visit https://doi.org/10.1017/S0950268815000102. click here to view supplementary material

## References

[1] Pegues D, Miller SI. *Salmonella* species, including *Salmonella* Typhi. In: Mandell G, Bennet J, Dolin R, eds. Principle and Practice of Infectious Diseases. Philadelphia: Churchill Livingstone Elsevier, 2010, pp. 2887–2903.

[2] Carrique-Mas JJ, Bryant JE. A review of foodborne bacterial and parasitic zoonoses in Vietnam. Ecohealth 2013; 10: 465–489. 24162798 10.1007/s10393-013-0884-9 PMC3938847

[3] Nga TV, The decline of typhoid and the rise of non-typhoid salmonellae and fungal infections in a changing HIV landscape: bloodstream infection trends over 15 years in southern Vietnam. Transactions of the Royal Society of Tropical Medicine and Hygiene 2012; 106: 26–34. 22137537 10.1016/j.trstmh.2011.10.004

[4] Thompson CN, Epidemiological features and risk factors of *Salmonella* gastroenteritis in children resident in Ho Chi Minh City, Vietnam. Epidemiology and Infection 2012; 141: 1–10. 23010148 10.1017/S0950268812002014 PMC3733064

[5] Vo ATT, Distribution of *Salmonella* enterica serovars from humans, livestock and meat in Vietnam and the dominance of *Salmonella* Typhimurium phage type 90. Veterinary Microbiology 2006; 113: 153–158. 16337754 10.1016/j.vetmic.2005.10.034

[6] Switt AI, Emergence, distribution, and molecular and phenotypic characteristics of Salmonella enterica serotype 4,5,12:i. Foodborne Pathogens and Disease 2009; 6: 407–415. 19292687 10.1089/fpd.2008.0213 PMC3186709

[7] Phan TT, Contamination of *Salmonella* in retail meats and shrimps in the Mekong Delta, Vietnam. Journal of Food Protection 2005; 68: 1077–1080. 15895745 10.4315/0362-028x-68.5.1077

[8] Van TT, Detection of *Salmonella* spp. in retail raw food samples from Vietnam and characterization of their antibiotic resistance. Applied and Environmental Microbiology 2007; 73: 6885–6890. 17766455 10.1128/AEM.00972-07 PMC2074948

[9] Thai TH, Antibiotic resistance profiles of *Salmonella* serovars isolated from retail pork and chicken meat in North Vietnam. International Journal of Food Microbiology 2012; 156: 147–151. 22497836 10.1016/j.ijfoodmicro.2012.03.016

[10] Ta YT, Prevalence of *Salmonella* on chicken carcasses from retail markets in Vietnam. Journal of Food Protection 2012; 75: 1851–1854. 23043836 10.4315/0362-028X.JFP-12-130

[11] Thai TH, Yamaguchi R. Molecular characterization of antibiotic-resistant *Salmonella* isolates from retail meat from markets in Northern Vietnam. Journal of Food Protection 2012; 75: 1709–1714. 22947480 10.4315/0362-028X.12-101

[12] Ta YT, Quantification, serovars, and antibiotic resistance of salmonella isolated from retail raw chicken meat in Vietnam. Journal of Food Protection 2014; 77: 57–66. 24405999 10.4315/0362-028X.JFP-13-221

[13] Carrique-Mas JJ, An epidemiological investigation of *Campylobacter* in pig and poultry farms in the Mekong delta of Vietnam. Epidemiology and Infection 2013; 142: 1425–1436. 24067502 10.1017/S0950268813002410 PMC4045178

[14] Arnold ME, Cook AJ. Estimation of sample sizes for pooled faecal sampling for detection of *Salmonella* in pigs. Epidemiology and Infection 2009; 137: 1734–1741. 19416556 10.1017/S0950268809002702

[15] Arnold ME, A comparison of pooled and individual bird sampling for detection of Salmonella in commercial egg laying flocks. Preventive Veterinary Medicine 2011; 99: 176–184. 21288584 10.1016/j.prevetmed.2010.12.007

[16] Carrique-Mas JJ, Davies RH. Sampling and bacteriological detection of *Salmonella* in poultry and poultry premises: a review. Revue Scientifique et Technique (International Office of Epizootics) 2008; 27: 665–677. 19284036 10.20506/rst.27.3.1829

[17] Skov MN, Evaluation of sampling methods for the detection of *Salmonella* in broiler flocks. Journal of Applied Microbiology 1999; 86: 695–700. 10212414 10.1046/j.1365-2672.1999.00715.x

[18] Arnold ME, Estimation of the sensitivity of environmental sampling for detection of *Salmonella* in commercial layer flocks post-introduction of national control programmes. Epidemiology and Infection 2014; 142: 1061–1069. 24020913 10.1017/S0950268813002173 PMC9151119

[19] Carrique-Mas JJ, Comparison of three plating media for the isolation of *Salmonella* from poultry environmental samples in Great Britain using ISO 6579:2002 (Annex D). Journal of Applied Microbiology 2009; 107: 1976–1983. 19558470 10.1111/j.1365-2672.2009.04386.x

[20] Anon. Antigenic Formulae of the *Salmonella* Serovars. 9th edn. WHO Collaborating Center for Reference and Research on Salmonella, 2007.

[21] Tennant SM, Identification by PCR of non-typhoidal *Salmonella* enterica serovars associated with invasive infections among febrile patients in Mali. PLoS Neglected Tropical Diseases 2010; 4: e621. 20231882 10.1371/journal.pntd.0000621 PMC2834738

[22] Clinical and Laboratory Standards Institute (CLSI). Performance standards for antimicrobial susceptibility testing, Twenty-First Informational Supplement, 2011, 100-S21, vol. 31, no. 1, pp. 42–58.

[23] Dohoo I, Martyn W, Stryhn H. Veterinary Epidemiologic Research. Charlottetown, Canada: AVC Inc., 2003.

[24] Hosmer D, Lemeshow S. 2000. Applied Logistic Regression, 2nd edn. John Wiley and Sons, Hoboken, USA.

[25] EFSA. Analysis of the baseline survey on the prevalence of *Salmonella* in holdings with breeding pigs in the EU, 2008: Part A: *Salmonella* prevalence estimates. EFSA Journal 2009; 7: 1377.

[26] EFSA. Report of the Task Force on Zoonoses Data Collection on the Analysis of the baseline survey on the prevalence of *Salmonella* in broiler flocks of *Gallus gallus*, in the EU, 2005–2006. EFSA Journal 2007; 98: 1–85.

[27] EFSA. Report of the Task Force on Zoonoses Data Collection on the Analysis of the baseline study on the prevalence of *Salmonella* in holdings of laying hen flocks of *Gallus gallus*. EFSA Journal 2007; 97.

[28] Minh PQ, A description of the management of itinerant grazing ducks in the Mekong river delta of Vietnam. Preventive Veterinary Medicine 2010; 94: 101–107. 20015558 10.1016/j.prevetmed.2009.11.011

[29] Tran TP, Prevalence of *Salmonella* spp. in pigs, chickens and ducks in the Mekong Delta, Vietnam. Journal of Veterinary Medical Science 2004; 66: 1011–1014. 15353859 10.1292/jvms.66.1011

[30] EFSA. The European Union Summary Report on trends and sources of zoonoses, zoonotic agents and food-borne outbreaks in 2012. EFSA Journal 2014; 12 (2): 3547 (312pp). 10.2903/j.efsa.2018.5500 PMC7009540 32625785

[31] Arnold ME, Carrique-Mas JJ, Davies RH. Sensitivity of environmental sampling methods for detecting *Salmonella* Enteritidis in commercial laying flocks relative to the within-flock prevalence. Epidemiology and Infection 2010; 138: 330–339. 19698210 10.1017/S0950268809990598

[32] Carrique-Mas JJ, Persistence and clearance of different *Salmonella* serovars in buildings housing laying hens. Epidemiology and Infection 2009; 137: 837–846. 19017427 10.1017/S0950268808001568

[33] Davies RH, Wray C. Mice as carriers of *Salmonella enteritidis* on persistently infected poultry units. Veterinary Record 1995; 137: 337–341. 8560683 10.1136/vr.137.14.337

[34] Phan TT, Prevalence of *Salmonella* spp. in rice-field rats in the Mekong delta, Vietnam. Journal of Veterinary Epidemiology 2005; 9: 85–88.

[35] Beloeil PA, Risk factors for *Salmonella* seroconversion of fattening pigs in farrow-to-finish herds. Veterinary Research 2007; 38: 835–848. 17903417 10.1051/vetres:2007034

[36] Garcia-Feliz C, Herd-level risk factors for faecal shedding of *Salmonella enterica* in Spanish fattening pigs. Preventive Veterinary Medicine 2009; 91: 130–136. 19539388 10.1016/j.prevetmed.2009.05.011

[37] Wray C. Salmonella in Domestic Animals. Oxon, UK: CABI Publishing, 2000.

[38] Bergeron N, Characterization of *Salmonella* Typhimurium isolates associated with septicemia in swine. Canadian Journal of Veterinary Research 2010; 74: 11–17. 20357952 PMC2801305

[39] Wagenaar JA, Hendriksen RS, Carrique-Mas J. Practical considerations of surveillance of *Salmonella* serovars other than Enteritidis and Typhimurium. Revue Scientifique et Technique (International Office of Epizootics) 2013; 32: 509–519. 24547654 10.20506/rst.32.2.2244

[40] Hauser E, Pork contaminated with *Salmonella* enterica serovar 4,[5],12:i:-, an emerging health risk for humans. Applied and Environmental Microbiology 2010; 76: 4601–4610. 20472721 10.1128/AEM.02991-09 PMC2901716

[41] Ha Thai T, Yamaguchi R. Molecular characterization of antibiotic-resistant salmonella isolates from retail meat from markets in northern Vietnam. Journal of Food Protection 2012; 75: 1709–1714. 22947480 10.4315/0362-028X.12-101

[42] EFSA. The European Union Summary Report on antimicrobial resistance in zoonotic and indicator bacteria from humans, animals and food in 2010. EFSA Journal 2012; 10: 233. 10.2903/j.efsa.2018.5182 PMC7009656 32625816

[43] Vo ATT, Antimicrobial resistance, class 1 integrons, and genomic island 1 in *Salmonella* isolates from Vietnam. PLoS ONE 2010; 5: e9440. 20195474 10.1371/journal.pone.0009440 PMC2829082

[44] Van TT, The antibiotic resistance characteristics of non-typhoidal Salmonella enterica isolated from food-producing animals, retail meat and humans in South East Asia. International Journal of Food Microbiology 2012; 154: 98–106. 22265849 10.1016/j.ijfoodmicro.2011.12.032

[45] Hopkins KL, Multiresistant Salmonella enterica serovar 4,[5],12:i:- in Europe: a new pandemic strain? Eurosurveillance 2010; 15: pii = 19 580. 20546690

[46] Deng X, Laboratory-based surveillance of non-typhoidal *Salmonella* infections in Guangdong Province, China. Foodborne Pathogens and Disease 2012; 9: 305–312. 22356574 10.1089/fpd.2011.1008

