# Supplementary material for: High levels of contamination and antimicrobial-resistant non-typhoidal Salmonella serovars on pig and poultry farms in the Mekong Delta of Vietnam
Source: Epidemiol Infect. 2015 Mar 17;143(14):3074–86. doi: 10.1017/S0950268815000102 (PMC4595858; doi:10.1017/S0950268815000102)
Supplement: Supplementary file 1 [file S0950268815000102sup001.docx]

Supplementary Table S1. Census of chicken, duck and pig farms, and number of farms sampled for NTS survey by farm size (Dong Thap, Mekong Delta, 2012).

|  | District | Chicken farms | | | Duck farms | | | Pig farms | | |
| --- | --- | --- | --- | --- | --- | --- | --- | --- | --- | --- |
|  |  | 20-50 | 51-100 | >100 | 50-200 | 201-1,000 | >1,000 | 5-20 | 21-50 | >50 |
| No. farms in census (No. sampled) | Cao Lanh | 7,430 (9) | 229 (10) | 46 (10) | 2,767 (8) | 1,178 (12) | 54 (10) | 302 (7) | 100 (9) | 14 (14) |
|  | Chau Thanh | 6,292 (10) | 150 (11) | 21 (10) | 1,875 (10) | 596 (10) | 35 (10) | 276 (11) | 433 (10) | 424 (9) |
|  | Hong Ngu | 4,840 (8) | 52 (11) | 8 (8) | 331 (7) | 249 (18) | 48 (5) | 320 (12) | 59 (7) | 3 (3) |
|  | Thanh Binh | 4,779 (9) | 106 (10) | 30 (11) | 1,233 (10) | 734 (10) | 78 (10) | 123 (7) | 28 (12) | 5 (3) |
| Sampling weights | Cao Lanh | 825.6 | 22.9 | 4.6 | 345.9 | 98.2 | 5.4 | 43.1 | 11.1 | 1.0 |
|  | Chau Thanh | 629.2 | 13.6 | 2.1 | 187.5 | 59.6 | 3.5 | 25.1 | 43.3 | 47.1 |
|  | Hong Ngu | 605.0 | 4.7 | 1.0 | 47.3 | 13.8 | 9.6 | 26.7 | 8.4 | 1.0 |
|  | Thanh Binh | 531.0 | 10.6 | 2.7 | 123.3 | 73.4 | 7.8 | 17.6 | 2.3 | 1.7 |
